# P-600. Efficacy of a Bivalent RSVpreF Vaccine in Older Adults Across a Second RSV Season

**DOI:** 10.1093/ofid/ofae631.798

**Published:** 2025-01-29

**Authors:** Edward E Walsh, John Woodside, Gonzalo Perez Marc, Daniel P Eiras, Qin Jiang, Conrado J Llapur, Yasushi Fukushima, Mika Rämet, Louis J Bont, Nazreen Hussen, Tarek Mikati, Michael Patton, Agnieszka Zareba, Kumar Ilangovan, Maria Maddalena Lino, Elena Kalinina, David Cooper, Kena A Swanson, Annaliesa S Anderson, Alejandra C Gurtman, Iona Munjal

**Affiliations:** University of Rochester, Rochester, NY; Pfizer, London, England, United Kingdom; i-trials, Buenos Aires, Ciudad Autonoma de Buenos Aires, Argentina; Pfizer, Inc., Pearl River, New York; Pfizer, London, England, United Kingdom; Hospital del Niño Jesús, San Miguel de Tucuman, Tucuman, Argentina; Fukuwa clinic, Chuo-ku, Tokyo, Japan; Tampere University, Tampere, Pirkanmaa, Finland; University Medical Centre Utrecht, Utrecht, Utrecht, Netherlands; Netcare Lakeview Hospital, Benoni, Gauteng, South Africa; 3. Pfizer, Inc., Vaccine Research & Development, Pearl River, New York; Pfizer, Vaccine Research and Development, Hurley, England, United Kingdom; Pfizer, London, England, United Kingdom; Vaccine Research and Development, Pfizer, USA, Raleigh, North Carolina; Pfizer, London, England, United Kingdom; Pfizer, London, England, United Kingdom; Pfizer, London, England, United Kingdom; Pfizer, London, England, United Kingdom; Pfizer, London, England, United Kingdom; Pfizer, London, England, United Kingdom; Pfizer Inc

## Abstract

**Background:**

In May 2023, the FDA approved RSVpreF [ABRYSVO™] for prevention of lower respiratory tract infection (LRTI) caused by RSV in adults ≥60 years of age. Here we present the vaccine efficacy (VE) data from the pivotal phase 3 global RENOIR study (NCT05035212) for an understanding of VE persistence through 2 RSV seasons and its impact on healthcare utilization (HCU).

**Figure 1:**
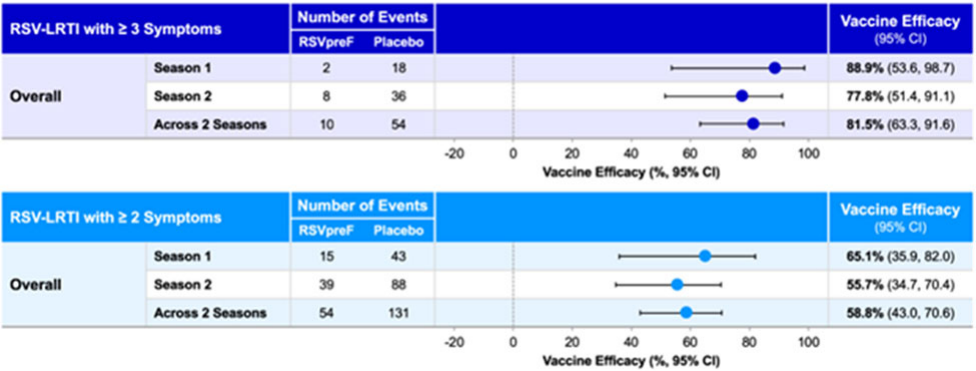
RSVpreF vaccine efficacy against LRTI-RSV through 2 RSV seasons

**Methods:**

Participants ≥60 years were randomized 1:1 to receive bivalent RSVpreF or placebo and were followed over two RSV seasons (Season 1 [S1]: N=36124; Season 2 [S2]: N=32223). VE was evaluated for RSV-LRTI with ≥3 symptoms (3+ LRTI-RSV), ≥2 symptoms (2+ LRTI-RSV), RSV-ARI, and by RSV subgroups, age, and at-risk conditions. HCU was evaluated by LRTI-RSV events that prompted any medically attended (MA) visit (hospitalization, primary care practitioner (PCP), urgent care (UC), emergency room (ER), specialist, other).

**Figure 2.**
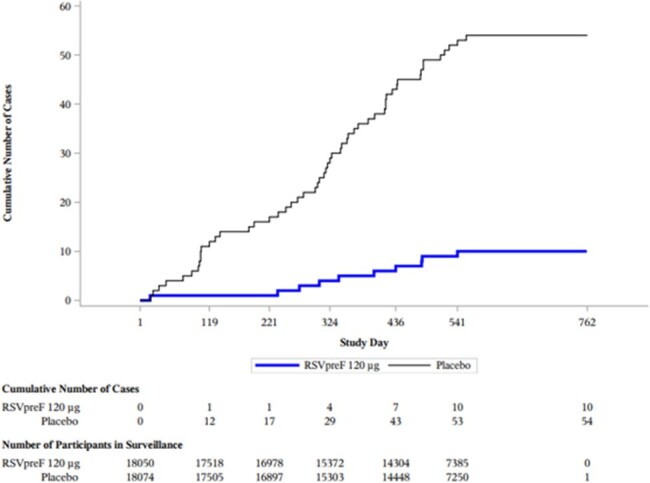
Cumulative case accrual curve from day of vaccination, first episode of LTRI-RSV with ≥3 symptoms from Day 15 through end of Season 2

**Results:**

The average acute respiratory illness (ARI) surveillance period within S1 and S2 was 7.1 and 7.6 months, respectively. Among the participants who contributed to the VE in S2, the average time from vaccination through the end of surveillance was 17.6 months. VE for 3+ LRTI-RSV was 88.9% (53.6, 98.7) in S1 and 77.8% (51.4, 91.1) in S2; VE across 2 seasons was 81.5% (63.3, 91.6). VE was ≥80% for both RSV A and B subgroups across S1 and S2. VE trended similarly by age, at-risk conditions, 2+ LRTI-RSV and RSV-ARI. VE for MA 3+ LRTI-RSV was 84.6 (32.0, 98.3) in S1, 72.0% (33.4, 89.8) in S2 and across 2 seasons was 76.3% (50.2, 89.9).

75% and 73% of all 3+ LRTI-RSV cases required a HCU visit in S1 and S2 respectively; placebo cases accounted for 87% of all 3+ LRTI-RSV related HCU visits in S1 and 78% in S2. Across both seasons, there were 4 hospitalizations: 1 RSVpreF compared to 3 placebo and 66 outpatient visits: 11 RSVpreF (ER = 9%, UC = 45%, PCP = 18%, specialist = 9%, other = 18%) compared to 55 placebo (ER = 18%, UC = 16%, PCP = 44%, specialist = 7%, other = 4%).

**Table 1.**
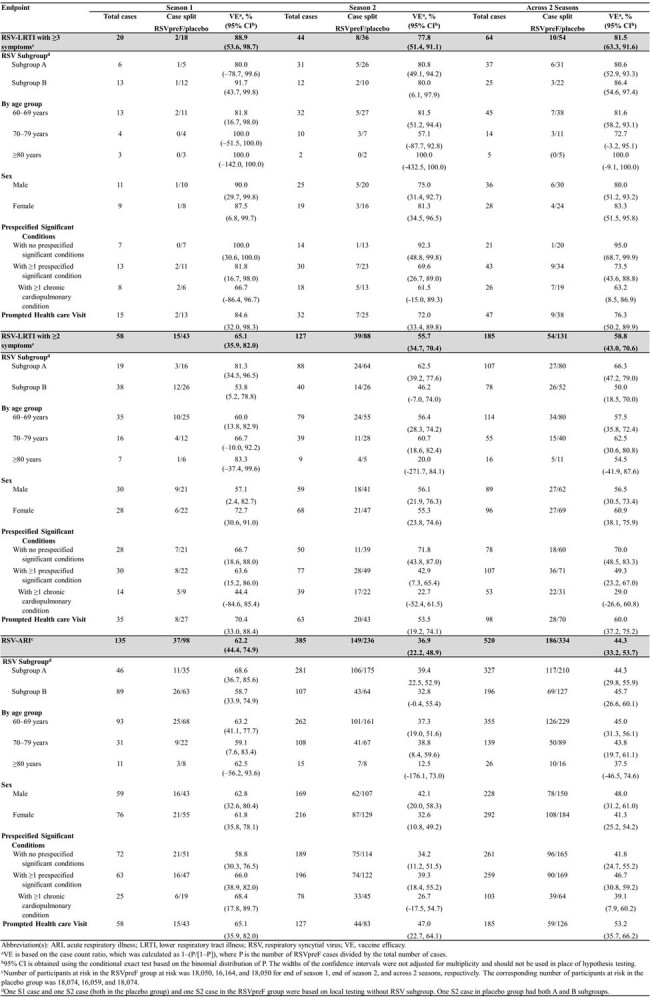
Vaccine efficacy of RSVpreF through 2 seasons, and across 2 seasons

**Conclusion:**

Bivalent RSVpreF remains efficacious against severe RSV-LRTI and continued to reduce RSV-related healthcare burden in older adults through a second RSV season. Consistent VE was demonstrated by age and at-risk individuals and across the RSV A and B subgroups supporting durable seasonal protection irrespective of RSV strain and the potential benefit to a bivalent RSV vaccine.

**Table 2:**
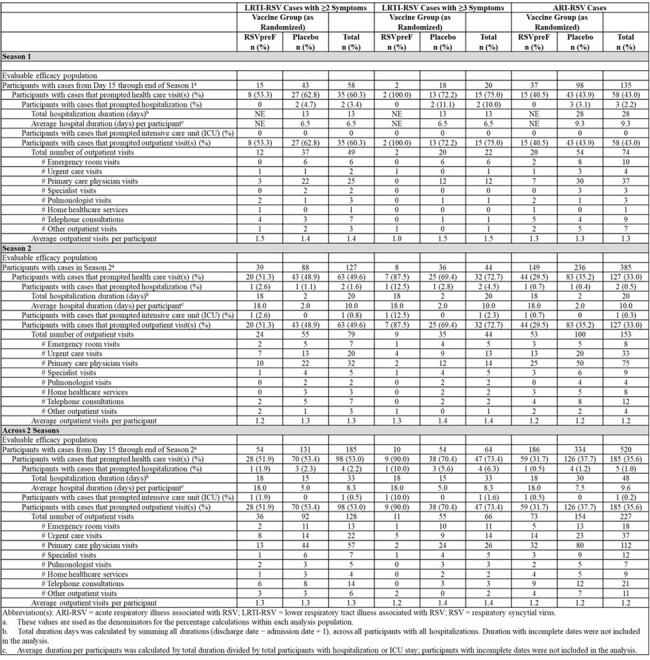
Healthcare Resource Utilization Associated With RSV Cases by RSV Season

**Disclosures:**

**Edward E. Walsh, MD**, Enanta: Advisor/Consultant|Enanta: Honoraria|GSK: Advisor/Consultant|Janssen: Advisor/Consultant|Merck: Advisor/Consultant|Merck: Grant/Research Support|Pfizer: Advisor/Consultant|Pfizer: Grant/Research Support **John Woodside, PhD**, Pfizer: Stocks/Bonds (Public Company)|Pfizer, Inc.: salary|Pfizer, Inc.: Stocks/Bonds (Public Company) **Gonzalo Perez Marc, MD**, Cassará: Expert Testimony|Cassará: Honoraria|Merck: Grant/Research Support|Moderna: Expert Testimony|Pfizer, Inc.: Advisor/Consultant|Pfizer, Inc.: Expert Testimony|Pfizer, Inc.: Grant/Research Support|Pfizer, Inc.: Clinical research|Sanofi: Grant/Research Support **Daniel P. Eiras, MD, MPH**, Pfizer. Inc: Employee|Pfizer. Inc: Stocks/Bonds (Private Company) **Qin Jiang, PhD**, Pfizer: Salary|Pfizer: Stocks/Bonds (Public Company) **Conrado J. Llapur, MD**, Pfizer, Inc: Grant/Research Support **Tarek Mikati, MD,MPH**, Pfizer, Inc.: salary|Pfizer, Inc.: Stocks/Bonds (Public Company) **Michael Patton, B.Sc.**, Pfizer: Employee|Pfizer: Stocks/Bonds (Public Company) **Agnieszka Zareba, MD PhD**, Pfizer, Inc.: salary|Pfizer, Inc.: Stocks/Bonds (Public Company) **Kumar Ilangovan, MD, MSPH, MMCi**, Pfizer, Inc.: salary|Pfizer, Inc.: Stocks/Bonds (Public Company) **Maria Maddalena Lino, PhD**, Pfizer: Stocks/Bonds (Private Company) **Elena Kalinina, PhD**, Pfizer, Inc.: Salary|Pfizer, Inc.: Stocks/Bonds (Public Company) **David Cooper, PhD**, Pfizer, Inc.: Employee|Pfizer, Inc.: Stocks/Bonds (Public Company) **Kena A. Swanson, Ph.D.**, Pfizer: Employee of Pfizer|Pfizer: Stocks/Bonds (Public Company) **Annaliesa S. Anderson, PhD**, Pfizer, Inc.: Employee|Pfizer, Inc.: Stocks/Bonds (Public Company) **Alejandra C. Gurtman, M.D.**, Pfizer, Inc.: Employee|Pfizer, Inc.: Stocks/Bonds (Public Company) **Iona Munjal, MD**, Pfizer: Salaried employee|Pfizer: Stocks/Bonds (Public Company)

